# Understanding the Impact of Obesity and Parental Blood Pressure in Identifying Optimal Hypertension Screening Group in Youth

**DOI:** 10.7759/cureus.74550

**Published:** 2024-11-27

**Authors:** Domagoj Koncar, Ana Kovacevic, Marijana Miler, Lavinia La Grasta Sabolic, Zivka Dika, Dora Softic, Bernardica Valent Moric

**Affiliations:** 1 Department of Pediatrics, Sestre milosrdnice University Hospital Center, Zagreb, HRV; 2 Department of Clinical Chemistry, Sestre milosrdnice University Hospital Center, Zagreb, HRV; 3 School of Medicine, Catholic University of Croatia, Zagreb, HRV; 4 Department of Nephrology, Arterial Hypertension, Dialysis and Transplantation, University Hospital Centre Zagreb, Zagreb, HRV; 5 School of Medicine, University of Zagreb, Zagreb, HRV

**Keywords:** ambulatory blood pressure monitoring, masked hypertension, obese children, parental hypertension, youth hypertension

## Abstract

Background

The rising incidence of hypertension (HTN) in pediatric patients imposes the need for its timely recognition by finding the optimal screening population. The goal of our study was to explore the ambulatory blood pressure (BP) parameters in selected groups of obese children and adolescents with different obesity levels and quantify the impact of parental hypertension (PH) on their blood pressure (BP) values.

Methods

This retrospective study included 176 obese Caucasian patients, 94 (53.4%) males, aged 6-18 years, who were divided based on their office blood pressure (OBP), body mass index (BMI) Z-score, and history of PH.

Results

Patients with PH had a significantly higher prevalence of masked hypertension (MH) and higher BMI (p=0.007 and p<0.001, respectively) compared to those with normotensive parents. There was no difference in whether HTN was of maternal or paternal origin, although the subjects with both hypertensive parents had higher diastolic blood pressure (DBP) parameters: office DBP (p=0.013), 24-hour DBP (p=0.017), and nighttime DBP (p=0.002). The multivariate regression analysis identified office systolic blood pressure (SBP) as a significant overall predictor of HTN (p<0.001), including the group with normotensive parents. In contrast, resting heart rate (HR) was an important predictor of HTN in subjects with PH (p=0.002). Additionally, a non-dipping BP pattern was predominantly observed in obese subjects, regardless of the degree of obesity (p=0.587).

Conclusion

Our results emphasize the importance of performing ambulatory blood pressure monitoring (ABPM) in obese children and adolescents, especially those with a history of PH. This group represents the target screening population for MH, which increases cardiovascular risk in this population when combined with obesity.

## Introduction

Hypertension (HTN) in the pediatric population has an increasing prevalence and is still not adequately recognized [1]. Growing evidence indicates the importance of recognizing high blood pressure (BP) in youth and its transfer to an adult HTN, which eventually leads to target organ damage (TOD) [2]. TOD begins silently; however, the research conducted by Yang et al. found outcomes such as left ventricular hypertrophy (LVH), elevated carotid intima-media thickness, and renal damage already at a young age [3]. Timely recognition and intervention already during pediatric care could reduce a huge medical burden regarding adult cardiovascular morbidity and mortality [4].

There is a clear association between increased office blood pressure (OBP) in youth and adverse cardiovascular outcomes later in life. However, relying solely on OBP provides an incomplete understanding of the overall risk. Ambulatory blood pressure monitoring (ABPM) is a more advanced tool for quantifying this relationship [5]. Following its success in adult HTN management, ABPM has become an indispensable method in assessing HTN in children and adolescents [6,7]. Comparing it to OBP, ABPM not only correlates better with the development of TOD but also provides more reproducible data and valuable insights into BP patterns during both daily activities and sleep [8]. Additionally, in obese children and adolescents, ABPM is particularly beneficial for predicting the risk of TOD in adulthood, given the higher prevalence of high-normal BP and masked hypertension (MH) in this group [9].

Obesity in children and adolescents is one of the most important health challenges in the 21st century, with its rising prevalence, especially in low- and middle-income countries [10]. Some studies have clearly shown a significant proportional effect of increasing body mass index (BMI) level on both OBP and ABPM values, indicating that obesity is not a binary condition [11,12].

Parental hypertension (PH) is often considered a variable that may influence BP levels in their offspring [13-15]. The findings are consistent regardless of whether ABPM was employed, confirming the familial clustering of elevated BP. However, studies focusing on obese children in this context remain scarce.

The primary goals of our study were to analyze ABPM in obese children and adolescents and quantify the impact of PH on ABPM parameters. Additionally, we aimed to determine whether obese youth and/or those with PH represent a key group for initiating HTN screening.

## Materials and methods

Subjects

The study was based on the retrospective data of 176 obese Caucasian patients, 94 (53.4%) males, aged 6-18 years (median: 14.3 years), who were admitted to the Department of Pediatrics at Sestre milosrdnice University Hospital Center in Zagreb, Croatia, primarily for the evaluation of obesity. The inclusion criteria consisted of obesity, properly measured OBP, a known parental history of HTN or normotension, and accurately performed ABPM. The exclusion criteria were acute illness at the time of ABPM, the use of medications that could interfere with BP, and the presence of endocrinological, renal, or congenital heart disease that could lead to a secondary HTN.

Ethical considerations

The study was approved by the Ethics Committee of Sestre milosrdnice University Hospital Center, Zagreb, Croatia (approval number: 251-29-11-21-03).

Methods

During the anthropometric assessment, subjects were dressed only in light indoor clothing. Body mass was measured using a standard balance scale with a precision of 0.1 kg, and body height was measured using a standard height board with a precision of 0.1 cm. A child was defined as obese if his or her BMI was at or above the 95th percentile for age and gender. BMI was presented as a standard deviation score based on the Kromeyer-Hauschild reference system [16]. The PH data relied on self-reported HTN or normotension in the parents.

OBP measurement was performed by an experienced healthcare worker using a clinically valid sphygmomanometer (DuraShock DS54 Thumbscrew, Welch Allyn, New York) with an appropriately sized cuff on the non-dominant arm. All subjects were seated in a quiet, calm room for 10 minutes prior to measurement. BP was measured three times at intervals of 3-5 minutes, and the average of the last two measurements was recorded. For simplicity, the term high-normal BP was not used; instead, we considered all subjects with OBP less than the 95th percentile for age, gender, and height (up to age 16) as office normotensive. In adolescents aged 16 or older, we applied the adult criteria defining OBP values below 140/90 mm Hg as normal [17].

ABPM was performed using a validated oscillometric device (Mobil-O-Graph M01100120, I.E.M.GmbH, Stolberg, Germany) with an appropriately sized cuff placed on the non-dominant arm. Daytime and nighttime measurement frequencies were set to 15-minute intervals from 07:00 to 22:00 and 30-minute intervals from 22:00 to 07:00. ABPM was performed during the workweek to assess BP in a regular daily rhythm. A minimum of 58 BP measurements (75% of total readings) were recorded over a 24-hour period, with at least one reading per hour. The reference values for mean 24-hour, daytime, and nighttime ABPM parameters (systolic blood pressure (SBP), diastolic blood pressure (DBP), mean arterial pressure, etc.) were taken from the relevant diagnostic thresholds [18].

Our observations were based on three main divisions. The first one was based on OBP, classifying patients as either normotensive or having high OBP. The second division considered PH, categorizing subjects as those with PH (one or both parents with known HTN) or without PH. Finally, the subjects were divided according to the BMI Z-score, forming two groups of equal size: Group 1 with a BMI Z-score of 1.70-2.69 and Group 2 with a BMI Z-score of 2.70-4.47.

Statistical analysis

The normality of data distribution was assessed using the D'Agostino-Pearson test. Due to the small sample size in some groups and the non-normal distribution of the data, non-parametric tests were used. The Mann-Whitney test was used to evaluate differences in parameters between groups. Categorical data and the proportions of different stages of continuous arterial BP monitoring between groups were analyzed using the chi-square test and the test of proportions. In addition, a multivariate regression analysis was performed to identify potential parameters for predicting HTN in patients. Patients' ages were presented as median and range (minimum to maximum), while the other variables were presented as median with interquartile range or as numbers and ratios. A p-value < 0.05 was considered statistically significant for all tests. A univariate regression analysis was performed to identify factors that could predict the diagnosis of hypertension in all patients and patients with parental hypertension. Following the univariate regression analysis, a multivariate analysis that included statistically significant predictors with p values < 0.05 was performed. Statistical analyses were performed using MedCalc software (version 11.5.1.0, Mariakerke, Belgium).

## Results

Our study included 176 obese patients, out of which 118 (67%) patients were office normotensive, and the rest had high OBP. In total, 74 (42%) patients had one or both parents with known HTN. Our patients with PH were significantly older (p=0.001) and had higher BMI (p<0.001) than those whose parents were not hypertensive. The clinical characteristics of the study population are presented in Table 1.

**Table 1:** Clinical characteristics of the study population *Statistically significant, p<0.05 BMI: body mass index, F: female, M: male, OBP: office blood pressure

Parameters (unit)	Without parental hypertension (N=102) (58%)	Parental hypertension (N=74) (42%)	P
Sex (M/F)	55/47	39/35	0.994
Age (years)	14 (6-18)	15 (7-18)	0.001*
BMI (kg/m^2^)	31.3 (28.6-34.8)	34.55 (31.5-39.9)	<0.001*
BMI Kromeyer Z-score	2.585 (2.20-2.98)	2.835 (2.60-3.30)	0.001*
Systolic OBP (mm Hg)	120 (110-140)	120 (115-140)	0.347
Diastolic OBP (mm Hg)	80 (70-81.25)	80 (70-80)	0.052

Analyzing ABPM parameters in these two groups, we found statistically significant differences in daytime systolic (p=0.003) and diastolic (p=0.014) BP, nighttime systolic (p=0.001) and diastolic (p=0.024) BP, and 24-hour systolic (p=0.004) and diastolic (p=0.013) BP, as well as daytime systolic (p=0.003) and diastolic (p=0.042) BP load. All these parameters were higher in subjects with PH (Table 2).

**Table 2:** Ambulatory blood pressure parameters in patients based on the presence of parental hypertension *Statistically significant, p<0.05 BP: blood pressure, DBP: diastolic blood pressure, SBP: systolic blood pressure

Parameters (unit)	Without parental hypertension (N=102) (58%)	Parental hypertension (N=74) (42%)	P
24-hour ambulatory BP			
SBP (mm Hg)	120 (116-129)	126.5 (121-133)	0.004*
DBP (mm Hg)	70 (65-74)	74 (68-76)	0.013*
Daytime ambulatory BP			
SBP (mm Hg)	122 (118-131)	129 (124-134)	0.003*
DBP (mm Hg)	72 (68-76)	75 (71-80)	0.014*
Nighttime ambulatory BP			
SBP (mm Hg)	114 (107-122)	118 (114-128)	0.001*
DBP (mm Hg)	62 (57-65)	63.5 (60-68)	0.024*
BP load			
Daytime SBP load	0.195 (0.11-0.46)	0.405 (0.21-0.54)	0.003*
Daytime DBP load	0.13 (0.07-0.24)	0.19 (0.09-0.33)	0.042*
Dipping			
SBP nocturnal dipping (%)	8.6 (4.4-11.0)	7.2 (3.0-10.0)	0.086
DBP nocturnal dipping (%)	15.9 (10.6-19.8)	14 (9.3-20.0)	0.619

**Figure 1:**
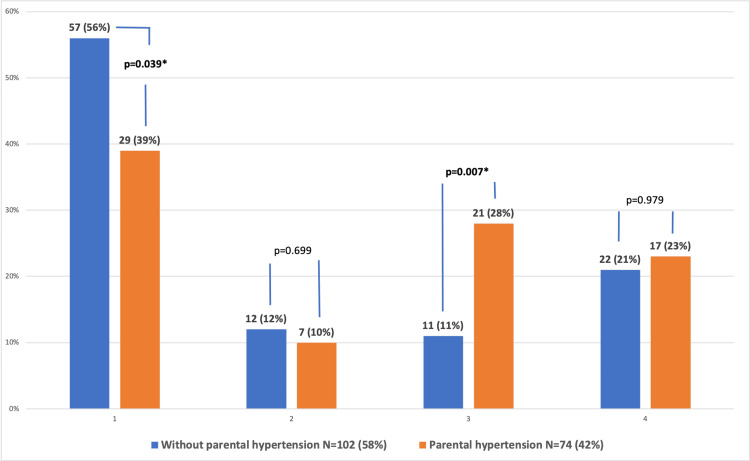
Influence of parental hypertension on different blood pressure categories *Statistically significant, p<0.05 1: normal blood pressure, 2: white coat hypertension, 3: masked hypertension, 4: hypertension

Further analysis did not show any difference in the prevalence of sustained HTN among these two groups (23% with PH versus 21% without PH, p=0.979). However, patients with PH had a significantly lower prevalence of normal OBP (39% versus 56% without PH, p=0.039) and a higher prevalence of MH (28% versus 11% without PH, p=0.007). A positive association was found between the PH and pathological ABPM category when the results of patients with normotension and the ones with either MH or sustained HTN were compared separately. There was no significant difference in white coat hypertension (WCH) prevalence (10% with PH versus 12% without PH, p=0.699) (Figure 1).

When analyzing our patients based on their obesity level, those who were mildly obese (BMI Z-score: 1.70-2.69) were more likely to have a normotensive parent (p=0.005). Furthermore, this group exhibited a higher prevalence of WCH (p=0.002) and a lower prevalence of MH (p<0.001) compared to the severely obese patients (Table 3).

**Table 3:** Influence of BMI and parental hypertension on ABPM parameters Group 1 represents obese patients with a BMI Z-score of 1.70-2.69 and Group 2 obese patients with a BMI Z-score of 2.70-4.47. *Statistically significant, p<0.05 ABPM: ambulatory blood pressure monitoring, BMI: body mass index, F: female, M: male

Parameters		Group 1 (N=88) (50%)	Group 2 (N=88) (50%)	P
Sex	F (number (%))	31 (35%)	51 (58%)	0.004*
	M (number (%))	57 (65%)	37 (42%)	0.004*
Parental hypertension	No (number (%))	61 (69%)	41 (47%)	0.005*
	Yes (number (%))	27 (31%)	47 (53%)	0.005*
ABPM category	Normal blood pressure (number (%))	44 (50%)	42 (48%)	0.909
	White coat hypertension (number (%))	16 (18%)	3 (3%)	0.002*
	Masked hypertension (number (%))	5 (6%)	27 (31%)	<0.001*
	Hypertension (number (%))	23 (26%)	16 (18%)	0.204

Although OBP levels of severely obese patients were comparable to those of mildly obese patients, they had significantly higher 24-hour systolic blood pressure (SBP) and diastolic blood pressure (DBP) (p=0.042 and p=0.021, respectively), as well as daytime SBP and DBP (p=0.033 and p=0.015, respectively). Additionally, severely obese patients showed greater daytime SBP and DBP load (p=0.049 and p=0.019, respectively), nighttime SBP and DBP load (p=0.002 and p=0.048, respectively), and nighttime SBP (p=0.024). Overall, 66.5% of patients were classified as non-dippers; however, there was no significant difference in the dipping status between these groups (p=0.587).

In all patients, the univariate logistic regression showed the statistical significance of the following parameters: resting heart rate (HR) (p<0.001), systolic OBP (p<0.001), diastolic OBP (p<0.001), 24-hour SBP (p<0.001), 24-hour DBP (p<0.001), daytime SBP (p<0.001), daytime DBP (p<0.001), daytime SBP load (p<0.001), daytime DBP load (p<0.001), nighttime SBP (p<0.001), nighttime DBP load (p<0.001), and nighttime SBP load (p<0.001). However, in the multivariate regression analysis, statistical significance was lost for most parameters. Statistically significant risk factors for HTN were female gender (p=0.008), younger age (p=0.006), systolic OBP (p<0.001), and daytime SBP load (p=0.006) with a high total area under the curve (AUC) value of 0.986 (95% confidence interval (CI): 0.953-0.998).

In patients with known PH, only resting HR (p=0.002) and daytime SBP load (p=0.002) remained statistically significant in the multivariate regression analysis with an AUC of 0.943 (95% CI: 0.860-0.985), as shown in Table 4.

**Table 4:** Regression model for predicting hypertension diagnosis in patients with a positive family history *Statistically significant, p<0.05 AUC: area under the curve, BMI: body mass index, BP: blood pressure, CI: confidence interval, DBP: diastolic blood pressure, F: female, OBP: office blood pressure, OR: odds ratio, SBP: systolic blood pressure

Parameters	Univariate regression analysis			Multivariate regression analysis		
	OR (95% CI)	P	AUC (95% CI)	OR (95% CI)	P	AUC (95% CI)
Sex (F)	0.26 (0.08-0.89)	0.032*	0.654 (0.535-0.761)	-	-	0.943 (0.860-0.985)
Age	0.76 (0.60-0.97)	0.030*	0.685 (0.567-0.788)	-	-	
BMI	0.87 (0.77-0.99)	0.029*	0.664 (0.544-0.769)	-	-	
Resting heart rate	1.09 (1.04-1.15)	<0.001*	0.828 (0.719-0.908)	1.13 (1.05-1.23)	0.002*	
OBP (systolic)	1.18 (1.08-1.28)	<0.001*	0.969 (0.898-0.996)	-	-	
OBP (diastolic)	1.14 (1.06-1.23)	0.001*	0.857 (0.753-0.929)	-	-	
24-hour SBP	1.25 (1.10-1.41)	0.001*	0.833 (0.729-0.910)	-	-	
24-hour DBP	1.24 (1.07-1.42)	0.003*	0.774 (0.662-0.863)	-	-	
Daytime SBP	1.22 (1.09-1.36)	0.001*	0.821 (0.715-0.901)	-	-	
Daytime DBP	1.20 (1.07-1.34)	0.002*	0.745 (0.630-0.839)	-	-	
Daytime SBP load	1549.47 (37.16-64608.93)	<0.001*	0.869 (0.770-0.936)	13111.12 (33.31-5160853.63)	0.002*	
Daytime DBP load	82.70 (5.84-1171.04)	0.001*	0.753 (0.639-0.846)	-	-	
Nighttime SBP	1.10 (1.03-1.17)	0.004*	0.743 (0.628-0.838)	-	-	
Nighttime DBP	1.10 (1.01-1.21)	0.032*	0.653 (0.533-0.760)	-	-	

While our study did not reveal any significant differences in HTN risk for obese offspring based on whether the HTN was maternal or paternal, it was noted that subjects with both hypertensive parents exhibited higher DBP parameters, including office DBP (p=0.013), 24-hour DBP (p=0.017), and nighttime DBP (p=0.002).

## Discussion

One of the key findings in our study is the positive correlation between the rising level of obesity and MH, particularly among subjects with PH compared to those with normotensive parents. Additionally, the analysis identified SBP load as an independent predictor of ABPM outcomes. Generally, obese youth show a non-dipping pattern, regardless of their obesity level. Furthermore, ABPM irregularities are proportional to the obesity level.

By the time this paper was completed, European Society of Hypertension (ESH) guidelines from 2016 were currently applicable [7]. These guidelines were also referenced in the recently published joint statement between HyperChildNET and the European Academy of Paediatrics regarding the diagnosis and management of HTN in youth, indicating that the diagnostic thresholds for HTN in children and adolescents have remained unchanged [17]. Meanwhile, multiple studies have compared the pediatric ESH criteria with those from the American Academy of Pediatrics (AAP). Genovesi et al. showed a significant difference in teenagers, with AAP recognizing HTN almost twice as often [19]. In their study, Kovačević et al. noted that only 36% of their patients, who were considered normotensive according to ESH guidelines, would be categorized as normotensive using the thresholds defined by AAP guidelines [12]. Similar discrepancies in classification were confirmed by studies from Furdela et al. [20] and Basaran et al. [21], with the latter indicating that the AAP guidelines were more effective in predicting LVH, particularly in normal-weight children under 15 years. Given that all our subjects were Croatian, we used European thresholds, which classify 67.6% of our 176 patients as office normotensive, while American guidelines drastically reduced that proportion to only 24.4%.

As previously mentioned, obesity undoubtedly correlates with HTN. Since all of our subjects were obese, comparisons were made based on their level of obesity, specifically the BMI Z-score. Similarly, Babinska et al. divided their subjects based on their BMI and noted an increase in ABPM irregularities with higher BMI, which aligns with our results [22]. Comparable results have also been demonstrated in various studies comparing obese with non-obese youth [11,23-25].

Non-dipping is defined as the absence of a physiological decrease in BP during the night, specifically a reduction of less than 10%. This phenomenon significantly contributes to overall cardiovascular risk. Consistent with the findings of Kovačević et al. [12] and Wühl et al. [18], we observed that obese youth do not show a dipping pattern, regardless of their BMI category. These results may suggest the existence of a BMI plateau that influences an individual's ability to lower nighttime BP. However, some studies have found that obesity in youth, unlike in adulthood, is not associated with dipping status; thus, we advocate for more extensive and well-designed studies in this area [11,25].

Another interesting ABPM component is the BP load, which represents the percentage of BP readings above the 95th percentile. In our analysis, daytime SBP load correlated with overall ABPM outcome in both the entire group and the PH subgroup (p<0.001 and p=0.002, respectively). Although earlier recommendations considered BP load an essential parameter in defining ABPM categories, several recent studies suggest otherwise, advocating for its elimination due to the demonstrated lack of prediction of TOD, particularly LVH [26,27].

The prevalence of MH in obese children and adolescents is reported to be between 9% and 16% [11,24]. Just as the understanding that the WCH phenomenon helps prevent over-diagnosis, recognizing MH uncovers a significant group of individuals who would otherwise remain undiagnosed. A key finding in our study regarding the classification of subjects by obesity level is the notable presence of MH. In our office normotensive group, 27.1% of subjects exhibited MH, rising to 64.3% in the severely obese subgroup, emphasizing the importance of performing ABPM in obese youth. These results are comparable to those of Valent Morić et al., who found MH in 31.6% of patients in the obese subgroup [25]. This data aligns with studies by Lurbe et al., which suggest that MH is much more common in overweight and obese patients than previously estimated [7]. However, that was not the case in the study by Stabouli et al., where none of the 23 obese patients (out of 85 patients included in the study) had MH, compared to 12.9% in the normal weight subgroup [23]. This discrepancy could be partially attributed to the relatively small sample size of obese patients included in their study.

The finding that MH is significantly more prevalent in obese youth with PH compared to those with normotensive parents (p=0.007) represents the key finding of our study. Therefore, these subjects should be considered as potential targets for HTN screening. Conversely, there was no difference in the HTN prevalence between children with PH and those with normotensive parents, nor was PH a predictor of sustained HTN in obese children. We hypothesize that our subjects with PH may remain more relaxed in front of the "white coat," ultimately revealing their true BP profile during a 24-hour evaluation. This could be explained by their familiarity with the BP measurement routine in their parents, leading to reduced anxiety when undergoing the same procedure at a doctor's office.

The recent study from Korea, involving a substantial sample of 3996 children and adolescents along with their 6394 parents, showed that the odds of HTN in offspring approximately doubled if one parent had HTN and quadrupled if both parents were hypertensive, at least based on OBP measurements [28]. Contrary to this finding, the results of our study did not demonstrate an association between OBP in children and their parents' HTN status. However, this relationship became evident when ABPM was used. This aligns with the findings from Gupta-Malhotra et al., who reported that the risk of HTN in children increased sevenfold when one parent had HTN and 14-fold when both parents were hypertensive, regardless of the parent's gender [14].

Similarly, a study by Alpay et al. using ABPM also emphasized the importance of PH, particularly maternal HTN, in determining the HTN risk in children [29]. Their study of 89 subjects with PH and 90 without, irrespective of obesity status, found an association only with maternal HTN. Furthermore, when examining the influence of the child's gender, females seemed to have relative protection against HTN [29]. In contrast, our study did not show a significant difference between maternal and paternal HTN in terms of risk. However, in subjects with both parents being hypertensive, we observed significantly higher DBP, both office, nighttime, and 24-hour readings, compared to those with only one hypertensive parent.

When examining the relationship between BP in youth and PH, Malbora et al. found that PH increases the risk of developing HTN, independent of BMI [13]. Zhao et al., in a study of 1288 high school freshmen, observed that while higher BMI was associated with a higher prevalence of HTN, PH was a stronger predictor of HTN, particularly in adolescents with normal weight [15]. Both studies emphasize the significance of PH but differ from our findings by not fully addressing the impact of rising BMI. Interestingly, Zhao et al. suggested that higher BMI might even offer some protective effect [15].

Although our results confirm the previously mentioned importance of ABPM, especially in obese youth, there remains hesitance regarding its broader implementation in routine HTN. The lack of devices and trained interpreters, along with uncertainties regarding appropriate indications, compliance, and optimal thresholds, continues to pose significant challenges for the future.

This study has several limitations. First, it relied on self-reported HTN data from parents who did not undergo the same thorough measurement as their children. Second, well-known risk factors for HTN, such as level of physical activity, diet, and socioeconomic background, were not considered. Additionally, the study was conducted at a single center with an ethnically uniform population, which may serve as a useful model for the local population but may have limited applicability to other contexts. Lastly, as a retrospective study, the findings may be subject to biases inherent in this design, such as reliance on past records and potential confounding factors that were not controlled for.

The major strength of our study lies in its novelty, as it focuses exclusively on obese children and adolescents, both office normotensive and hypertensive, who underwent ABPM and have known parental BP status.

## Conclusions

In conclusion, our study demonstrates a positive association between the increasing levels of obesity and MH, particularly in subjects with PH. We did not find any significant differences in HTN risk among obese offspring based on whether their mothers or fathers had HTN. However, subjects with both hypertensive parents exhibited higher DBP parameters. A non-dipping pattern was predominantly observed in our obese subjects, regardless of the severity of obesity.

Our findings highlight the importance of performing ABPM in obese children and youth, especially those with PH, representing them as an ideal screening population for MH. This is important, as MH is recognized as an additional risk factor that, alongside obesity, enhances cardiovascular risk and the development of TOD in this vulnerable population.

## References

[1] Koebnick C, Black MH, Wu J (2013). The prevalence of primary pediatric prehypertension and hypertension in a real-world managed care system. J Clin Hypertens (Greenwich).

[2] Juhola J, Oikonen M, Magnussen CG (2012). Childhood physical, environmental, and genetic predictors of adult hypertension: the cardiovascular risk in young Finns study. Circulation.

[3] Yang L, Yang L, Zhang Y, Xi B (2018). Prevalence of target organ damage in Chinese hypertensive children and adolescents. Front Pediatr.

[4] Pedrinelli R, Ballo P, Fiorentini C (2012). Hypertension and acute myocardial infarction: an overview. J Cardiovasc Med (Hagerstown).

[5] O'Brien E, Parati G, Stergiou G (2013). European Society of Hypertension position paper on ambulatory blood pressure monitoring. J Hypertens.

[6] Urbina E, Alpert B, Flynn J (2008). Ambulatory blood pressure monitoring in children and adolescents: recommendations for standard assessment: a scientific statement from the American Heart Association Atherosclerosis, Hypertension, and Obesity in Youth Committee of the Council on Cardiovascular Disease in the Young and the Council for High Blood Pressure Research. Hypertension.

[7] Lurbe E, Agabiti-Rosei E, Cruickshank JK (2016). 2016 European Society of Hypertension guidelines for the management of high blood pressure in children and adolescents. J Hypertens.

[8] Stergiou GS, Alamara CV, Salgami EV, Vaindirlis IN, Dacou-Voutetakis C, Mountokalakis TD (2005). Reproducibility of home and ambulatory blood pressure in children and adolescents. Blood Press Monit.

[9] Di Bonito P, Licenziati MR, Morandi A (2022). Screening for hypertension in young people with obesity: feasibility in the real life. Nutr Metab Cardiovasc Dis.

[10] (2024). World Health Organization: Obesity and overweight. https://www.who.int/news-room/fact-sheets/detail/obesity-and-overweight.

[11] Lurbe E, Invitti C, Torro I (2006). The impact of the degree of obesity on the discrepancies between office and ambulatory blood pressure values in youth. J Hypertens.

[12] Kovačević A, Vidatić I, Škorić I, Valent Morić B (2023). Does the body mass index category influence ambulatory blood pressure parameters in office normotensive obese children?. Pediatr Cardiol.

[13] Malbora B, Baskin E, Bayrakci US, Agras PI, Cengiz N, Haberal M (2010). Ambulatory blood pressure monitoring of healthy schoolchildren with a family history of hypertension. Ren Fail.

[14] Gupta-Malhotra M, Hashmi SS, Barratt MS, Milewicz DM, Shete S (2018). Familial aggregation of first degree relatives of children with essential hypertension. Blood Press.

[15] Zhao W, Mo L, Pang Y (2021). Hypertension in adolescents: the role of obesity and family history. J Clin Hypertens (Greenwich).

[16] Kromeyer-Hauschild K, Wabitsch M, Kunze D (2001). Percentiles for the body mass index for children and adolescents using various German samples (Article in German). Monatsschr Kinderheilkd.

[17] Lurbe E, Mancia G, Calpe J (2023). Joint statement for assessing and managing high blood pressure in children and adolescents: Chapter 1. How to correctly measure blood pressure in children and adolescents. Front Pediatr.

[18] Wühl E, Witte K, Soergel M, Mehls O, Schaefer F (2002). Distribution of 24-h ambulatory blood pressure in children: normalized reference values and role of body dimensions. J Hypertens.

[19] Genovesi S, Parati G, Giussani M (2020). How to apply European and American guidelines on high blood pressure in children and adolescents. A position paper endorsed by the Italian Society of Hypertension and the Italian Society of Pediatrics. High Blood Press Cardiovasc Prev.

[20] Furdela V, Pavlyshyn H, Kovalchuk T, Haliyash N, Luchyshyn N, Kozak K, Hlushko K (2022). Prevalence of arterial hypertension among Ukrainian students: the comparison of European and American guidelines. Pediatr Endocrinol Diabetes Metab.

[21] Basaran C, Kasap Demir B, Tekindal MA (2022). Re-evaluating hypertension in children according to different guidelines: a single-center study. Hypertens Res.

[22] Babinska K, Kovacs L, Janko V, Dallos T, Feber J (2012). Association between obesity and the severity of ambulatory hypertension in children and adolescents. J Am Soc Hypertens.

[23] Stabouli S, Kotsis V, Papamichael C, Constantopoulos A, Zakopoulos N (2005). Adolescent obesity is associated with high ambulatory blood pressure and increased carotid intimal-medial thickness. J Pediatr.

[24] So HK, Yip GW, Choi KC, Li AM, Leung LC, Wong SN, Sung RY (2016). Association between waist circumference and childhood-masked hypertension: a community-based study. J Paediatr Child Health.

[25] Valent Morić B, Jelaković B, Vidatić I, Trutin I, Jelaković A, Stipančić G (2020). Ambulatory blood pressure profile in office normotensive obese children: prevalence of masked hypertension and impact of parental hypertension. J Pediatr Endocrinol Metab.

[26] Sharma AP, Altamirano-Diaz L, Mohamed Ali M, Stronks K, Kirpalani A, Filler G, Norozi K (2021). Diagnosis of hypertension: ambulatory pediatric American Heart Association/European Society of Hypertension versus blood pressure load thresholds. J Clin Hypertens (Greenwich).

[27] Hamdani G, Mitsnefes MM, Flynn JT (2021). Pediatric and adult ambulatory blood pressure thresholds and blood pressure load as predictors of left ventricular hypertrophy in adolescents. Hypertension.

[28] Jang S, Kim ST, Kim YK, Song YH (2023). Association of blood pressure and hypertension between parents and offspring: the Korea National Health and Nutrition Examination Survey. Hypertens Res.

[29] Alpay H, Ozdemir N, Wühl E, Topuzoğlu A (2009). Ambulatory blood pressure monitoring in healthy children with parental hypertension. Pediatr Nephrol.

